# Lights, fiber, action! A primer on *in vivo* fiber photometry

**DOI:** 10.1016/j.neuron.2023.11.016

**Published:** 2023-12-15

**Authors:** Eleanor H. Simpson, Thomas Akam, Tommaso Patriarchi, Marta Blanco-Pozo, Lauren M. Burgeno, Ali Mohebi, Stephanie J. Cragg, Mark E. Walton

**Affiliations:** 1 Department of Psychiatry, Columbia University Medical Center, New York, NY, USA; 2 New York State Psychiatric Institute, New York, NY, USA; 3 Department of Experimental Psychology, University of Oxford, Oxford, UK; 4 Wellcome Centre for Integrative Neuroimaging, University of Oxford, Oxford, UK; 5 Institute of Pharmacology and Toxicology, University of Zürich, Zürich, Switzerland; 6 Neuroscience Center Zürich, University and ETH Zürich, Zürich, Switzerland; 7 Department of Neurology, University of California, San Francisco, San Francisco, CA, USA; 8 Department of Physiology, Anatomy and Genetics, University of Oxford, Oxford, UK; 9 Aligning Science Across Parkinson’s (ASAP) Collaborative Research Network, Chevy Chase, MD, USA; 10 Present address: Department of Biology, Stanford University, Stanford, CA 94305, USA

## Abstract

Fiber photometry is a key technique for characterizing brain-behavior relationships *in vivo*. Initially, it was primarily used to report calcium dynamics as a proxy for neural activity via genetically encoded indicators. This generated new insights into brain functions including movement, memory, and motivation at the level of defined circuits and cell types. Recently, the opportunity for discovery with fiber photometry has exploded with the development of an extensive range of fluorescent sensors for biomolecules including neuromodulators and peptides that were previously inaccessible *in vivo*. This critical advance, combined with the new availability of affordable “plug-and-play” recording systems, has made monitoring molecules with high spatiotemporal precision during behavior highly accessible. However, while opening exciting new avenues for research, the rapid expansion in fiber photometry applications has occurred without coordination or consensus on best practices. Here, we provide a comprehensive guide to help end-users execute, analyze, and suitably interpret fiber photometry studies.

## A BACKGROUND TO FIBER PHOTOMETRY AND OVERVIEW OF THIS PRIMER

Our understanding of how the brain works continues to be propelled by the development of methods that enable researchers to examine neural mechanisms during behavior. One such technique is fiber photometry. Initially, it was primarily used to report calcium dynamics as a proxy for neural activity via genetically encoded indicators in behaving animals.^1–3^ However, with the development of fluorescent sensors for numerous biomolecules including extracellular ligands (neurotransmitters and modulators) and intracellular signaling molecules that were previously inaccessible *in vivo*, interest in the technique has exploded. Increasingly, fiber photometry is seen as the technique of choice to measure neurotransmitter dynamics *in vivo* in rodents.

**Figure 1.**
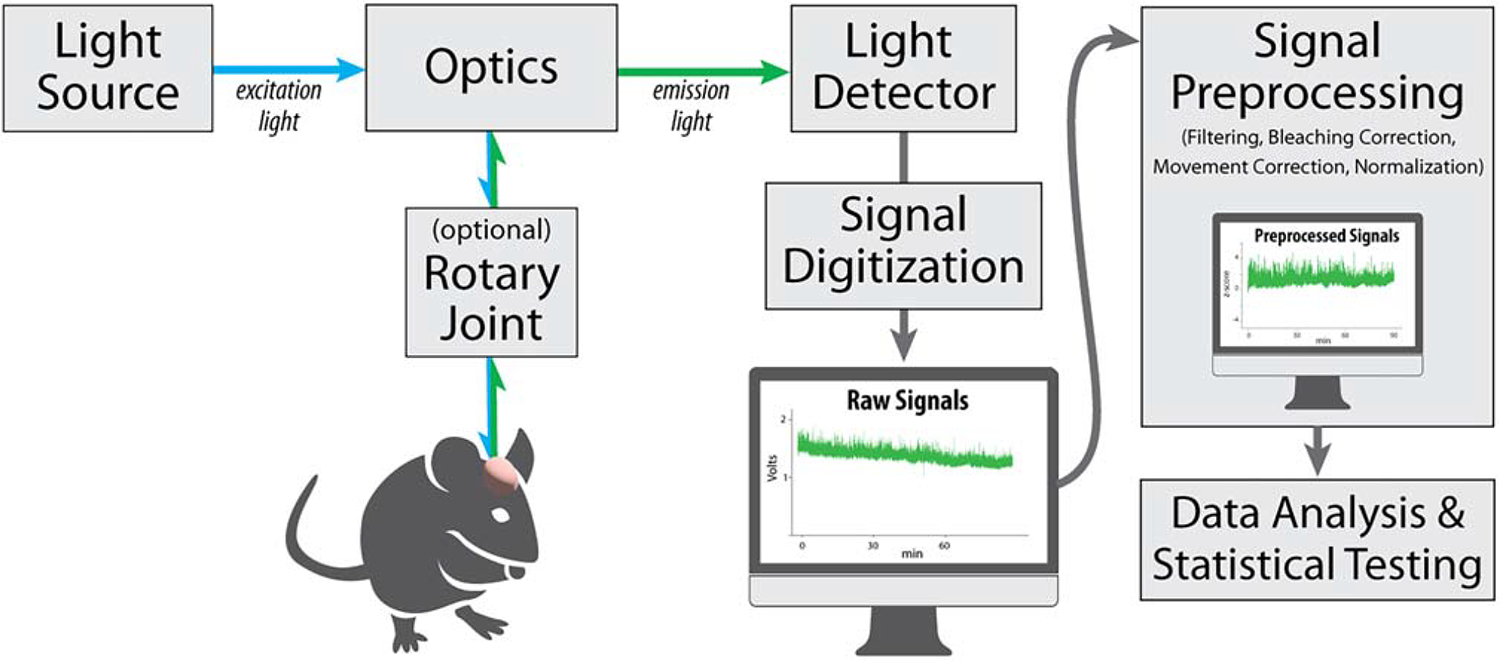
Schematic of the setup of a generic rodent *in vivo* fiber photometry experiment

Fiber photometry is an optical technique in which light is used to trigger and measure fluctuations in fluorescence that arise from conformational change to an expressed biosensor (Figure 1). Briefly, excitation light of a specific wavelength is delivered through an implanted optical fiber, and emitted fluorescence is returned via the same fiber to a photodetector. A digital optical intensity signal is then generated that is presumed to reflect the relative amount of the target bound sensor at the tip of the fiber. As the detected signal comes from the tissue around the fiber tip, which may range from 50 to 400 μm, it reflects a regional, or “bulk,” readout. However, because biosensors are genetically encoded, their expression can be directed to defined circuits and/or cell types where they can be stable for several weeks to months. No other *in vivo* technique permits repetitive recordings over such long periods of time. This technique has already enabled unprecedented insights into how population activity in particular cell groups relates to components of complex behavior including movement, memory, motivation, appetitive and aversive learning, and more.^3–6^

The rapid increase in popularity of fiber photometry is a testament to its many practical advantages over other approaches for *in vivo* monitoring of neural signals in behaving animals (see Table 1). Unlike electrophysiology, fiber photometry can straightforwardly provide signals with molecular and cellular specificities. It can have higher spatial resolution and much higher temporal resolution than typical microdialysis experiments, which usually have sample rates on the order of ~10s of minutes (although see Zhang et al.,^7^ Wang et al.,^8^ and Ngernsutivorakul et al.^9^ for recent specialized advances that have brought microdialysis resolution down to the sub-minute range). Concurrent within-subject recordings of dopamine using photometry and standard microdialysis highlight differences in observable temporal dynamics.^10^ Compared with cyclic voltammetry, photometry can offer greater sensitivity for some analytes/environments. For example, measuring dopamine *in vivo* with chronically implanted carbon-fiber microelectrodes using fast scan cyclic voltammetry (FSCV) is more challenging in the dorsal compared with the ventral striatum, but this is not the case when using dopamine sensors.^11^ Photometry also provides access to molecules for which there are no electrochemical methods available. For example, *in vivo* fluctuations in acetylcholine were previously inferred from amperometric measures of choline,^12^ and it is now known that such signals can be confounded by phasic oxygen dynamics.^13^ Fluorescent biosensors now provide a more direct (and faster) measure of acetylcholine, e.g., Chantranupong et al.^14^ In terms of its practical application, photometry also has several other benefits as follows: the surgical procedures are much less invasive than for microscopy-based approaches, and the use of flexible, lightweight optical fibers to record signals are less restrictive to natural behavior than many other preparations. An increasing number of “plug-and-play” systems are becoming available, many at relatively low cost, thus creating opportunities for diverse communities of researchers to conduct experiments to characterize brain-behavior relationships at scale. Another advantage of fiber photometry over other *in vivo* techniques is the relatively low size and complexity of the raw data compared with electrophysiology, two-photon, or mini-microscope imaging. The processing of photometry data requires a solid understanding of the technique and careful consideration of possible confounding factors, as outlined in this primer, but there is no computationally demanding spike-sorting or single-cell extraction required. Such low dimensionality means there are no barriers or limitations to data sharing among groups, which, if broadly adopted, will foster replication and reproducibility.Arguably, the greatest excitement around fiber photometry is generated by its potential for novel applications. Dozens of different biosensors have already been developed and, theoretically, sensors for any native molecule can be created. Using multi-channel systems, multiple probes, sensors of different wavelengths, and combinations of transgenic lines and viral vectors, there are countless opportunities for multiplexed and multi-layered experiments. Linking neurotransmitter release to real-time effects on downstream circuits and examining the coordination of transmitter release or activity across projections sites, are just some of the possible opportunities. Fiber photometry also offers an unparalleled opportunity to monitor fluctuations in biomolecules or physiological events for multiple hours and analyze the fluctuations across different time scales, ranging from sub-second to tens of minutes. This feature may be of critical value in systems neuroscience when the fundamental mechanisms of information encoding, or computation are not yet known.

Balanced with this promise are potential methodological pitfalls, which render interpreting the data challenging. In theory, or in a test tube or flow cell, dose-response curves make the relationship between ligand concentration and fluorescence intensity appear straightforward. However, measuring ligand-modulated fluorescence *in vivo*, where photometric signals are neither linear nor absolute measures, is more complicated. Signals are influenced by native factors, including local fluctuations in pH and hemodynamics,^15–17^ and technical factors including the expression level and localization of the sensors, excitation wavelengths, potential photobleaching, and the stability of the optical path, each of which will be discussed. This is complicated by the fact that methods of data collection and analysis are legion, and all too often are minimally described, making it problematic to compare or integrate findings across different labs.

Therefore, here, we provide a comprehensive guide to the choices end-users will need to make when collecting, analyzing, and interpreting information using fiber photometry. The goal of this primer was to help the scientific community leverage the transformative potential that fiber photometry offers.

## SENSOR SELECTION FOR *IN VIVO* PHOTOMETRY

A wide range of genetically encoded fluorescent sensors have been developed and validated using *in vivo* fiber photometry. These include multiple classes of sensors with distinct molecular designs and spectral properties.^18,19^ Each of these indicators can be used to parse out dynamic changes in a specific biomolecule or physiological event, ideally with little to no cross-talk or influence from other aspects of neural activity. Examples of biomolecules or physiological events that can be monitored using *in vivo* photometry range from intracellular calcium,^2,20,21^ to intracellular signaling molecules like cyclic-AMP (cAMP)^22,23^ and protein kinase A (PKA),^24,25^ to membrane voltage,^26,27^ and to extracellular ligands, such as endogenous neurotransmitters,^28–31^ neuromodulators,^32–40^ or exogenously administered drugs^41^ (Table 2). Some of these events are cell intrinsic (e.g., calcium, voltage, cAMP, and PKA) and are sometimes utilized as a proxy to gauge neural activity. Others represent molecules that are released into extracellular space and can sometimes diffuse across micrometer-scale distances (e.g., neurotransmitters and neuromodulators). These two distinct classes of events encode information differently. In the first case, the integration of multiple events occurs within the confines of each cell of interest; in the second case, endogenous receptors on the surface of target cells receive and interpret spatiotemporal patterns of multiple neurotransmitters.

All these sensors share the following two underlying features: they are fully genetically encoded and are composed of a sensing domain and a fluorescent reporter domain. In general, the sensing domain generates a conformational change in response to ligand binding or changes in membrane voltage that is then converted into a photometry-detectable fluorescent readout by the fluorescent reporter domain. As a result, sensing domains determine the kinetics, affinity, and, where applicable, ligand specificity of the sensor. Sensing domains can be made of calmodulin and a Ca^2+^/calmodulin-binding peptide (e.g., M13,^66–68^ ckkp,^46,48^ and ENOSP^42^), as in the case of the widely used GCaMP-type calcium sensors.^20^ Alternatively they can be made of cAMP-binding domains,^23,63^ kinase-specific phosphorylation motifs fused to their recognition domains,^24,25^ rhodopsin voltage-sensing domains (VSDs),^65^ periplasmic-binding proteins (PBPs),^28^ or G-protein-coupled receptors.^32,52^ In specific cases, such as for catecholamines, the ligand selectivity of the sensor may not be sufficient to unambiguously assign the nature of the detected signal, particularly in brain areas where relative abundance of the two molecules is largely skewed. For example, detecting norepinephrine in subregions of the basal ganglia is difficult because both neuromodulators are present but dopamine by far dominates. In such cases, specific experiments with dual-color recordings involving pairs of dopamine and norepinephrine probes may be needed to address this issue.

The fluorescent reporter domains determine the nature of the output signal (i.e., wavelength, intensity, or lifetime) and the dynamic range of the sensor. Typically, these are made of a circularly permuted fluorescent protein (e.g., circularly permuted green fluorescent protein [cpGFP]), which provides a rapid and direct intensiometric readout, chiefly due to the modulation of its chromophore’s microenvironment and protonation state.^69^ Other reporter systems generate a change in fluorescence intensity or lifetime during Forster resonance energy transfer between a fluorescent protein donor and an acceptor (electrochromic-fluorescence resonance energy transfer [FRET] sensors^65^ or FRET-fluorescence lifetime imaging microscopy [FLIM] sensors^24,25,64^). In particular, “FLIM”-based sensors hold great potential for fiber photometry recordings. Although intensity-based photometry measures the amounts of photons received from a sample, its lifetime-based counterpart focuses on how the photons are distributed in time after excitation with a pulsed laser. Fluorescence lifetime measures are thus independent from the total number of collected photons, making it insensitive to variation in sensor expression levels, laser power, scattering and reabsorption of the tissue, and optical losses in the detection apparatus.^69–72^ Therefore, FLIM-based sensors could provide more robust and reliable signals for comparison across animals and time and to better protect the readout from potential unwanted sources of variability.^73^ This technique has not yet widely taken off, partly because ready-to-use FLIM-adapted photometry hardware is not yet commercially available (see section on advanced hardware features).

A comparison of many of these tools in terms of peak spectral excitation and emission properties, ligand affinity, sensor dynamic range (i.e., maximal response to ligand), kinetic parameters, the animal models in which these tools have been deployed, and considerations related to ligand-buffering has been extensively reviewed elsewhere.^18,19,37,74,75^ Here, we focus on other important considerations for selecting the most appropriate sensor for *in vivo* fiber photometry experiments. In particular, we discuss the use of control wavelengths that have been spectrally defined to be insensitive to changes in analyte levels, sensor-specific control experiments, and challenges and opportunities for multiplexing sensors.

### Choice of sensor-specific controls

Interpretation of *in vivo* fiber photometry signals can be challenging, especially in cases where the observed signals are similar in amplitude to background noise. The inclusion of appropriate controls will mitigate the risk of data misinterpretation. A common approach is to generate a negative control signal—i.e., a signal acquired under the same conditions as the experimental signal of interest, but which is not expected to vary with the physiological process being measured. This can be used to support the conclusion that the observed signals faithfully represent “real” fluctuations in the process under investigation and/or to attempt to correct for confounding variation in the signal, such as movement artifacts or photobleaching (see section pre-processing).

Depending on the type of sensor used, different types of negative controls have been implemented. A simple method to obtain a negative control signal is to use a “stable” fluorescent protein, such as GFP,^43^ YFP,^3^ mcherry,^53^ or tdTomato,^43^ either expressed independently or in combination with the sensor if the two can be spectrally resolved.^43^ This is an effective way of generating a control signal for movement artifact correction but limits the possibility of multiplexing the sensor with other probes or optogenetic tools (see below). Another approach is to illuminate the sensor at its *isosbestic* wavelength, i.e., the wavelength at which sensor fluorescence does not vary with changes in ligand concentration. Exciting the sensor at this wavelength therefore results in emitted fluorescence that provides a stable reference signal. A list of known isosbestic points of *in vivo* photometry-compatible sensors is shown in Table 2. Isosbestic wavelengths vary substantially across different sensors, over a wavelength range of 350–440 nm. Appropriate excitation light sources and filter sets are required for the specific sensor(s) isosbestic wavelength (see section hardware for fiber photometry).

For sensor responses that rely on a ligand-binding event, control signals can be obtained from sensors in which mutations have been introduced within the sensing domain to abolish ligand binding (Table 2). Mutant non-ligand-binding control sensors are particularly important for understanding photometry readout when a ligand-binding sensor detects ligands that vary on the same slower time courses or low amplitudes as physiological artifacts (such as pH or hemodynamic changes). This approach has been extensively utilized for several sensors engineered from GPCRs, facilitated by the large amount of structural and mutagenesis data available from previous pharmacological studies. Sensing domain mutations have also been utilized to generate control PBP-based sensors,^33^ a cAMP-based sensor,^23^ and a FRET-FLIM sensor,^25^ demonstrating the general viability of such an approach (for details see Table 2). Unlike coexpression of a spectrally resolvable fluorescent protein or isosbestic controls, these methods must be performed in separate animals or brain regions and thus may not fully recapitulate the same conditions of the experimental recordings.

**Figure 2.**
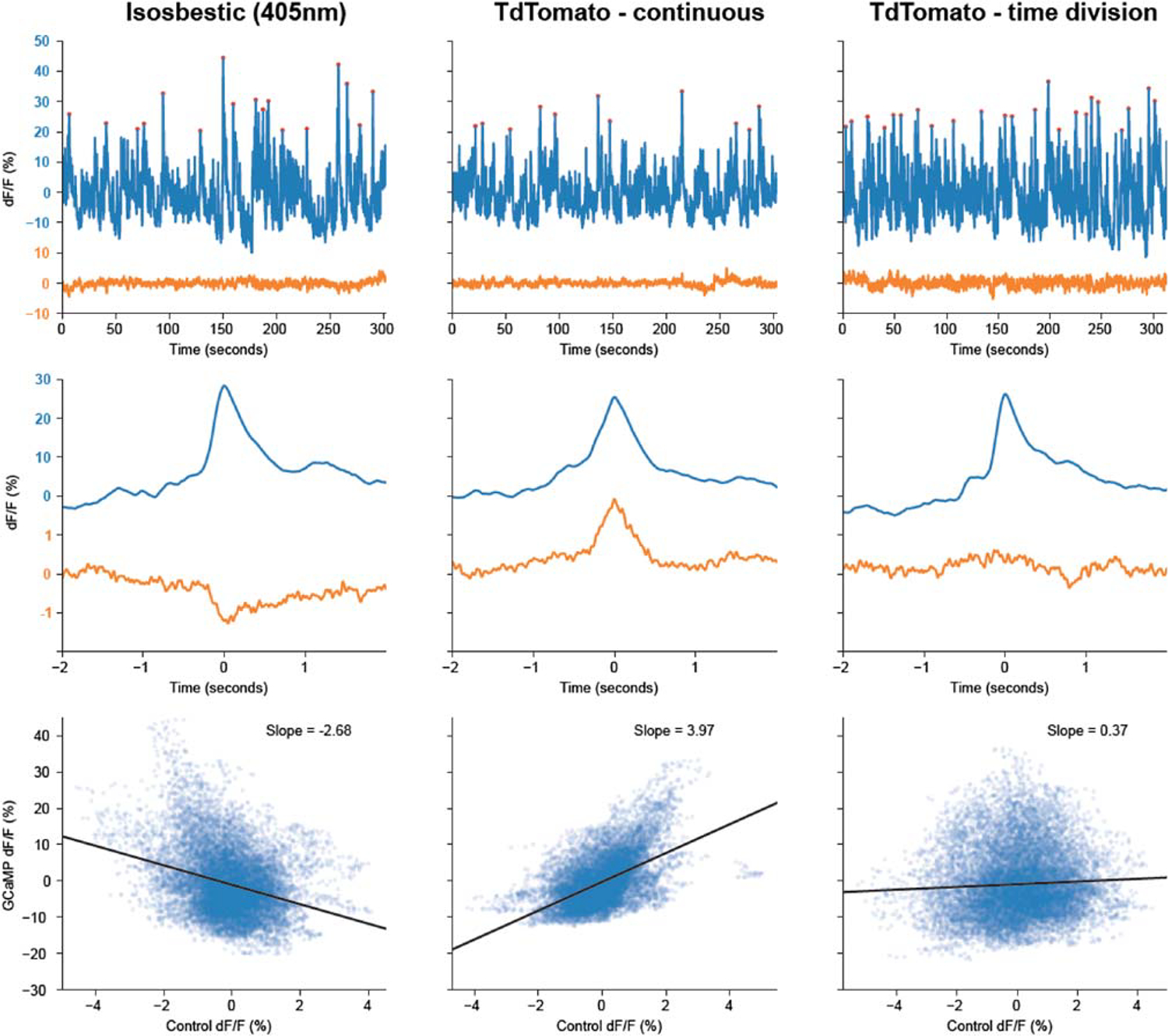
Control signals and their caveats Comparison of different movement control signal methods, acquired in the same subject and experimental setting. GCaMP6f and tdTomato were expressed in VTA dopamine neurons, and signals were recorded during exploration of novel objects. In all recordings the GCaMP signal was measured using 470 nm excitation and 525 nm emission filters. Three different methods were used to generate the movement control signal: left panels, using “isosbestic” illumination of the GCaMP (405 nm excitation and 525 nm emission), using time-division illumination (alternatively pulsing the 405 and 470 nm LEDs) to separately acquire the control and GCaMP signal; middle panels, by measuring the tdTomato fluorescence (560 nm excitation and 640 nm emission) with the LEDs for both the GCaMP and tdTomato channels on continuously; right panels, by measuring the tdTomato fluorescence, but using time-division illumination (alternately pulsing the 470 and 560 nm LEDs). Top row. Example GCaMP signal (blue) and control signal (magenta) over a 5-min recording. Middle row. Average GCaMP and control signals aligned on the peaks in the GCaMP signal indicated by the red dots in top row. Bottom row. Scatterplot of the GCaMP signal against the control signal, with linear fit whose slope is indicated on the figure. Note that the isosbestic control signal has significant negative bleed-through of the GCaMP signal, due to 405 nm excitation not exactly matching the isosbestic point for GCaMP6f. This is evident as a negative peak in the event-aligned average and results in a strong negative correlation between the control and GCaMP channel. The tdTomato-continuous signal shows positive bleed-through of the GCaMP signal, due to overlap of the GCaMP emission spectra with the emission filter on the tdTomato channel, resulting in strong positive correlation between the signals. As variation in both these control signals is dominated by signal bleed-through, they could not be used to estimate movement artifacts in the signal channel. Using time-division illumination for the tdTomato channel greatly reduces cross-talk, as the GCaMP is not excited when the tdTomato signal is acquired, resulting in no evidence of signal bleed-through in the event-aligned traces, and a weak positive correlation between the channels consistent with a contribution only from small movement artifacts. Note also that the slope of the linear fit is <1, indicating that the movement artifacts in the GCaMP signal are smaller in dF/F terms than those in the tdTomato channel, consistent with a larger movement-insensitive autofluorescence contribution to the GCaMP channel due to the shorter wavelength illumination.

There are two important potential factors to be aware of when using a negative control signal (Figure 2). First, the relative contribution of sensor/fluorophore fluorescence and autofluorescence (from optical component and brain tissue) will in general be different for control and sensor signals, due to differences in excitation/emission wavelengths and/or fluorophore brightness. Typically, shorter wavelength excitation light results in a larger autofluorescence contribution, and as a large area, diffuse light source, autofluorescence is usually less affected by movement artifacts than sensor fluorescence. Autofluorescence will also not necessarily photobleach at the same rate as sensor fluorescence. Additionally, as light absorption by brain tissue is greater at shorter wavelengths, the volume of tissue from which signal is acquired will vary as a function of excitation and emission wavelength.^76,77^ Fluctuations in the control channel therefore will not necessarily have the same amplitude (in dF/F) as those in the sensor channel caused by the same mechanism.

Second, physiological signal variation in the sensor channel can bleed-through into the control channel. When a control fluorophore is used, bleed-through can occur due to the overlap of emission spectra with the sensor. Significant bleed-through from green indicators (e.g., GCaMP) to red control channels can occur if continuous illumination is used, but this can largely be eliminated by using modulated excitation light (see section hardware for fiber photometry; Figure 2). With an isosbestic control, bleed-through of physiological signals will occur if the excitation wavelength used does not accurately match the isosbestic point of the indicator. This may result in either positive or negative contamination of the control channel by the physiological signal. As movement artifacts or other confounding signals are typically small relative to sensor fluorescence changes, even a small amount of bleed-through from sensor to control channels can end up dominating variation in the control channel at behaviorally relevant frequencies (Figure 2), preventing accurate estimation of motion artifacts or other confounds (see pre-processing section).

Depending on the specific experimental design, additional control experiments may be required. A unique and useful feature of GPCR-based sensors is their intrinsic sensitivity to drugs targeting the receptor subtype they are based upon. Systemic administration of sensor-specific antagonists can drastically lower evoked sensor response during *in vivo* recordings.^32,35,36,38,52,57^ With careful planning, this approach can be used to obtain within-animal control recordings that can help qualify the nature of the observed signals. An important caveat of this approach is that drugs often have pleiotropic effects on an animal’s physiological functions, both central and peripheral. Thus, knowledge of the effects of the chosen drug on the animal’s physiology will aid interpretation. Factors to consider include direct and indirect effects on the neuromodulatory pathway under investigation (e.g., in the case of drugs affecting neuromodulator reuptake or autoreceptor mechanisms^40^) and alterations to other parameters, which may affect photometry readouts. For example, receptor antagonists that alter intracellular pH or produce hemodynamic changes could result in artifactual signals.

### Multiplexing

Some of the most exciting and useful *in vivo* applications of fiber photometry involve spectral multiplexing. Spectrally resolvable sensors may be expressed in the same brain area and excited via the same optic fiber to determine the relationship between multiple dynamic factors, e.g., calcium activity and neuromodulator release from the same or different genetically identified populations. Sensors may also be combined with fluorescence-based actuators to interrogate input-output relationships. The availability of a large color palette of optogenetic actuators^78,79^ and a growing color palette of optogenetic sensors^21,27,33,34,46,48^ enables an ever-increasing number of mix-and-match applications. For successful examples, see section current opportunities.

Multiplexing options are currently somewhat restricted at present by the limited availability of spectrally resolvable sensors for distinct aspects of neural activity since most sensors are based on GFP (see control sensors available in Table 2). In the near future, further expansion of the color palette of sensors based on fluorescent proteins with excitation shifted to red, far-red, or near-infrared wavelengths, along with the development of new photometry systems equipped for recording at these wavelengths, will make it possible to achieve spectral multiplexing of 3 or more fluorescence-based tools simultaneously.

### Biosensor delivery

In addition to selecting biosensor(s) with desired intrinsic properties, the method of biosensor delivery will impact the obtained signal, qualitatively and quantitatively. A biosensor may be constitutively or conditionally expressed in transgenic mouse strains. Transgenic expression of calcium sensors in some reporter mice can alter physiology^75^; hence, care must be taken to test for such undesired effects. More typically, biosensors are expressed episomally from viral vectors which provide a multiplicity of options. First, the choice of viral serotype may affect the spatiotemporal dynamics of the reported signal because serotypes differ in their tropism,^80–83^ the cell types they preferentially infect and therefore the density, cell type, neuronal class, and subsequently the subcellular localization of biosensor presentation governing distances between ligand and sensor.^11,84^ Next, within the viral construct, the choice of promoter that drives expression of the sensor can further restrict the cell-type specificity of expression. Due to vector size constraints, only a limited number of promoter-targeted constructs have been successfully developed. A more tractable way to direct expression is by injecting a conditional (e.g., Cre-dependent) construct into a transgenic mouse line expressing Cre recombinase under a cell-type-specific promoter. Using a viral vector that undergoes anterograde or retrograde transport allows for monitoring biosensor activity in the soma or axons of a specific projection. Combining these methods allows projection and cell-type specificity, for example, monitoring calcium dynamics in mesoaccumbal^11^ or striatonigral^84^ dopamine axons.

Whichever delivery approach is taken, the sensor expression level must be considered. If the viral titer is very high, a GPCR-based receptor may theoretically saturate the membrane or large quantities of fluorescent proteins may be detrimental to some sensitive cells.^85–87^ The intensity of expression within cells and the density of expressing cells or axons will impact the signal-to-noise ratio and therefore the effective dynamic range of the sensor. Comparisons of the tropism and efficacy of different adeno-associated virus (AAV)-based vectors have been reviewed elsewhere.^81,88^ One tool available to help identify an appropriate serotype, promoter, and titer for AAV vectors for a specific experiment is the AAV Data Hub hosted by Addgene (a popular source for viral vectors). Despite the influence that virus serotypes, promoters, and titers have on photometry data, these details are not consistently reported in publications. It would be helpful if they were.

## HARDWARE FOR FIBER PHOTOMETRY

A user’s choice of photometry system design depends on the technical capabilities required for their particular experiments. Within the given requirements, the time, effort, expertise, and money required to get a system working are important considerations. Currently, there are several companies selling photodetector-based or camera-based fiber photometry systems that are virtually plug-and-play. Alternatively, labs may design and build their own custom systems, making significant cost savings.^89,90^ A popular in-between approach that requires less time and expertise, but still affords flexibility, is to build a system by integrating a few different modular components. An advantage to buying or building a CCD, CMOS, or sCMOS camera-based system is that they can be used to collect data from fiber bundles, allowing simultaneous measurements from many separate sites and/or animals without amplifying the expense of costly photodetectors. However, cameras may be slower, less sensitive, or noisier than some photodetectors, which may be an important consideration, depending on the application. Here, we first note hardware factors that must be considered to ensure compatibility with common types of experimental design. We then introduce hardware options for advanced and emerging fiber photometry applications.

### Experiment-specific hardware considerations

#### Spectral channels

Most photometry systems record fluorescence signals from one or more discrete spectral channels defined by an excitation wavelength and emission wavelength (in practice both will be a range not single wavelength). Many systems incorporate a channel with excitation at ~470 nm and emission at ~520 nm for use with green fluorescent sensors. Additional channels allow for simultaneous measurement of multiple sensors with different excitation and emission spectra, and/or inclusion of a control channel to correct for movement artifacts (see section pre-processing). A typical dual-color photometry system uses 470 nm and 565 nm filters for the excitation light source for green and red fluorophores, respectively. Emission filters at ~520 and ~590 nm are then used to direct the fluorescence signal from each fluorophore to separate photodetectors. The optical hardware required for dual systems is more complicated and expensive. For experiments involving only a single sensor, a common way of obtaining a control signal is a channel that uses the same emission wavelength as the main channel (simplifying the hardware), but a different excitation wavelength chosen to be at the isosbestic point of the chosen sensor (see also section choice of sensor-specific controls).

To differentiate fluorescence excited at different wavelengths but emitted at the same wavelength, e.g., for an isosbestic control, excitation light is modulated to separate the emission evoked by each light source in either *time* (by alternately turning on each light source in turn) or *frequency* (by sinusoidally modulating each light source at a different frequency). These two solutions are typically referred to as time-division or frequency-division illumination, respectively. The recorded signal is then processed to demodulate (i.e., separate) the emission evoked by each excitation wavelength. These methods can also be useful even when spectral channels have different emission wavelengths because fluorescent sensors typically have broad emission spectra that can cause bleed-through between channels. Time- or frequency-division illumination can greatly reduce this issue by enabling both excitation and emission spectra to contribute to channel separation (Figure 2).

#### Signal bandwidth

The signal bandwidth is the frequency range of signals the system can record, typically from DC (0 Hz) to a maximum frequency. The signal bandwidth is determined both by the light detector hardware, and, if modulated excitation light is used, the filtering needed to demodulate the signals. For many applications, signal bandwidth is unlikely to be a limiting factor because fluorescence signals from most sensors are relatively slow (>>1 ms rise time), and signals will typically be low-pass filtered well below the system’s signal bandwidth during pre-processing to reduce noise. However, some specialist applications like membrane voltage sensors or fluorescent lifetime imaging involve much faster signals, making the system bandwidth an important consideration.

#### Sensitivity/noise level

Noise is inherent in any acquisition system and determines the smallest signals that can be accurately recorded. System sensitivity is most critical for experiments where the details of the biology result in very weak fluorescent signals, but sensitivity is desirable for all experiments as reduced noise allows lower excitation light power, and hence less photobleaching, for a given signal-to-noise (S/R) ratio.

Manufacturers of photodetectors typically report the *noise equivalent power* (NEP) of the device, given in watts per square root of hertz (W/√Hz). NEP is defined as the input signal power that gives a S/R of 1 after filtering the output to reduce its bandwidth to 1 Hz, which allows a meaningful comparison of noise levels between systems with different bandwidths. In contrast, camera manufacturers report noise using different measures that have not been standardized, making it difficult to compare across devices. Ideally, vendors of plug-and-play photometry systems with photodetectors or cameras would report the NEP for the complete signal path, from optical input to digitized output, but in practice, different vendors report different and not always very informative measures of system sensitivity, making meaningful comparisons difficult.

#### Rotary joints

A rotary joint (fiber-optic commutator) between the photometry system and the animal allows for freely moving experiments to be undertaken without the optic fiber getting twisted. Unfortunately, it currently appears to be very difficult to manufacture robust and reliable fiber-optic rotary joints that have the very low signal variation required for photometry at low cost. Some designs work better than others. For example, motor-assisted multi-channel pigtailed rotary joints offer superior stability, although this can add a substantial expense to the setups. One option is to simply not use a rotary joint but instead use a long patch cord to minimize constriction and twisting. An alternative approach offered in some commercial systems (that could also be custom built) is to mount all optical components including light sources and photodetectors, below an electrical rotary joint, such that the entire optical system rotates with the animal and optical signals do not need to pass through a rotary joint.

For experiments involving behavioral measures that are incompatible with tethering, some commercial wireless fiber photometry systems have been developed. However, some of these system designs are too large for mice, are limited to a single fiber, and comprise a single LED source and detector for GFP-like sensors only, lacking a control channel (see choice of sensor-specific controls section). To alleviate this problem, a dual-wavelength wireless platform was recently described.^91^

#### Multimodal experiments

For experiments that combine photometry with optogenetics, in addition to the spectral channels for acquiring signals, the system must be configured to allow light for optogenetic stimulation to be delivered through the same fiber. A suitable system may be purchased pre-configured or custom-built using published methods papers (e.g., Sych et al.,^92^ Qi et al.,^93^ and Formozov et al.^94^). Photometry systems compatible with simultaneous electrophysiology recordings are also commercially available or can be custom built (e.g., Patel et al.^95^).

### Advanced hardware features

Recently, some exciting applications of fiber photometry have emerged to address limitations, resolve confounding factors, or expand sampling dimensions associated with typical fiber photometry. These applications require specialist hardware with concomitant increases in cost and technical complexity. We anticipate that with further hardware development, these applications will become more popular so we briefly describe them here.

#### Spectrally resolved photometry

Spectrally resolvable sensors can be multiplexed, but if one sensor is markedly brighter than the other, there may be significant cross-talk between the two sensors. To overcome this limitation, Meng et al.^43^ developed spectrally resolved fiber photometry. Rather than recording signals at discrete wavelengths using a photodetector or camera, a spectrometer is used to record the full emission spectrum. By using known concentrations of fluorophores with different emission wavelengths, the contributions of each fluorophore to the collected spectrograms can be determined and used to generate a spectral linear unmixing algorithm. Although this technique requires more complex pre-processing of the data, it potentially allows better separation of sensors with different fluorophores and other sources of signal variability. For example, Zhang et al.^15^ used spectral fiber photometry to estimate the effects of changes in hemoglobin concentration on photometry signals. Oxy- and deoxy-hemoglobin have different absorption coefficients at the wavelengths typically used in fiber photometry, affecting the accuracy of photometry data. Using spectral fiber photometry, Zhang et al.^15^ accounted for, and corrected, changes in GCaMP6f responses in the somatosensory cortex that were driven by changes in blood oxygen level.

#### Depth-resolved photometry

Fiber photometry is typically done using flat-cut fibers. A flat-cut fiber allows fluorescence collection from the tissue immediately below the fiber face, estimated to be ~200 μm for a 200 μm fiber.^77,96^ By contrast, depth-resolved fiber photometry^97^ uses tapered fibers to collect light from a larger range (up to 2 mm) along the fiber axis. Furthermore, this method can be used to record from multiple sites using a single tapered fiber. The application uses galvanometric mirrors to systematically project laser beams into the tapered fibers at different angles resulting in laser beams exiting at distinct fiber locations. Recording depth is then resolved using a time-division multiplexing scheme. Another advantage of tapered fibers is that they minimize tissue damage compared with flat-cut fibers. However, depth-resolved fiber photometry requires more sophisticated hardware, which increases the system’s price and makes it more complicated to assemble in individual labs. At the time of writing this article, only one commercial solution is available for depth-resolved fiber photometry.

#### FLiP

Sensors that report changes in fluorescence lifetime provide an absolute measurement of ligand binding, which simplifies making comparisons across sessions and subjects compared with signals obtained with intensiometric sensors but require specialized hardware. An example setup for single fiber fluorescence lifetime photometry (FLiP) consists of a laser that provides ~50-Mhz-pulsed illumination, filters that separate emission from excitation light before focusing the fiber face on a high-speed photomultiplier tube that is connected to a time-correlated single photon counting board, which detects the time delay between the pulsed excitation and the photon detection by the photomultiplier tube. Such systems have been used to perform FLiP measures of multiple sensors to report real-time biochemical changes *in vivo.*^24,64^

## DATA PRE-PROCESSING AND ANALYSIS

Fiber photometry data are typically first *pre-processed* to remove noise and artifacts from the signals and convert them into meaningful units for comparison across recordings and subjects. The processed signals are then *analyzed* to understand how they covary with other experimental variables such as behavior. Next, we unpack each step in order.

There are many software options for implementing these steps. Commercial software is typically limited, and whether using pre-assembled hardware or a custom-built system, many users require more flexibility. Those with programming experience may write their own custom software in their language of choice. Alternatively, there are open-source packages that require little programming experience. Some versions are streamlined for handling data generated by specific commercial setups.^98^ Of the two most popular open-source photometry software packages, pMAT^99^ is more user-friendly, and GuPPY^100^ has the most flexibility (see Marquardt^101^ for detailed comparison). An advantage of all open-source software packages is that users benefit from pre-written code and can efficiently adapt it as needed. For others to understand and evaluate results generated using custom or modified code, the code must be publicly available in a well-documented form.

### Pre-processing

Pre-processing involves multiple steps. It is very helpful to observe raw data and intermediate stages of processing, both at the timescale of the entire session and zoomed into short time windows. This helps identify artifacts or problems with the data and generates an understanding of how each processing step modifies the signals. Small changes to the pre-processing should not result in qualitative changes to the results; examining the data after each processing step will help diagnose problems should they occur.

Before describing processing steps in detail, it is useful to consider the components that make up the photometry signal and drive its variation. Light that reaches the detector comes from multiple sources: fluorescence from the indicator(s), autofluorescence from the patch cord and other optical components, autofluorescence from brain tissue, and potentially bleed-through of excitation light and/or background illumination. Depending on the biological preparation and hardware, fluorescence from the indicator may be only a small fraction of the total light detected. Likewise, multiple sources contribute variation to the signal: change in fluorescence of the indicator due to the physiological process(es) of interest (e.g., fluctuation in calcium or neuromodulator concentration), photobleaching of the indicator, photobleaching of the patch cord and optical components, physical movement, local changes in blood flow, and noise from the detector hardware. Preprocessing aims to correct confounding sources of variability to yield an accurate assessment of the physiological signal of interest.

There is substantial variability across studies in preprocessing methods used, and there has been little systematic comparison of different approaches. Our aim here was to outline widely used approaches, comment on their benefits and limitations, and highlight areas where a systematic comparison of different methods would be useful.

**Figure 3.**
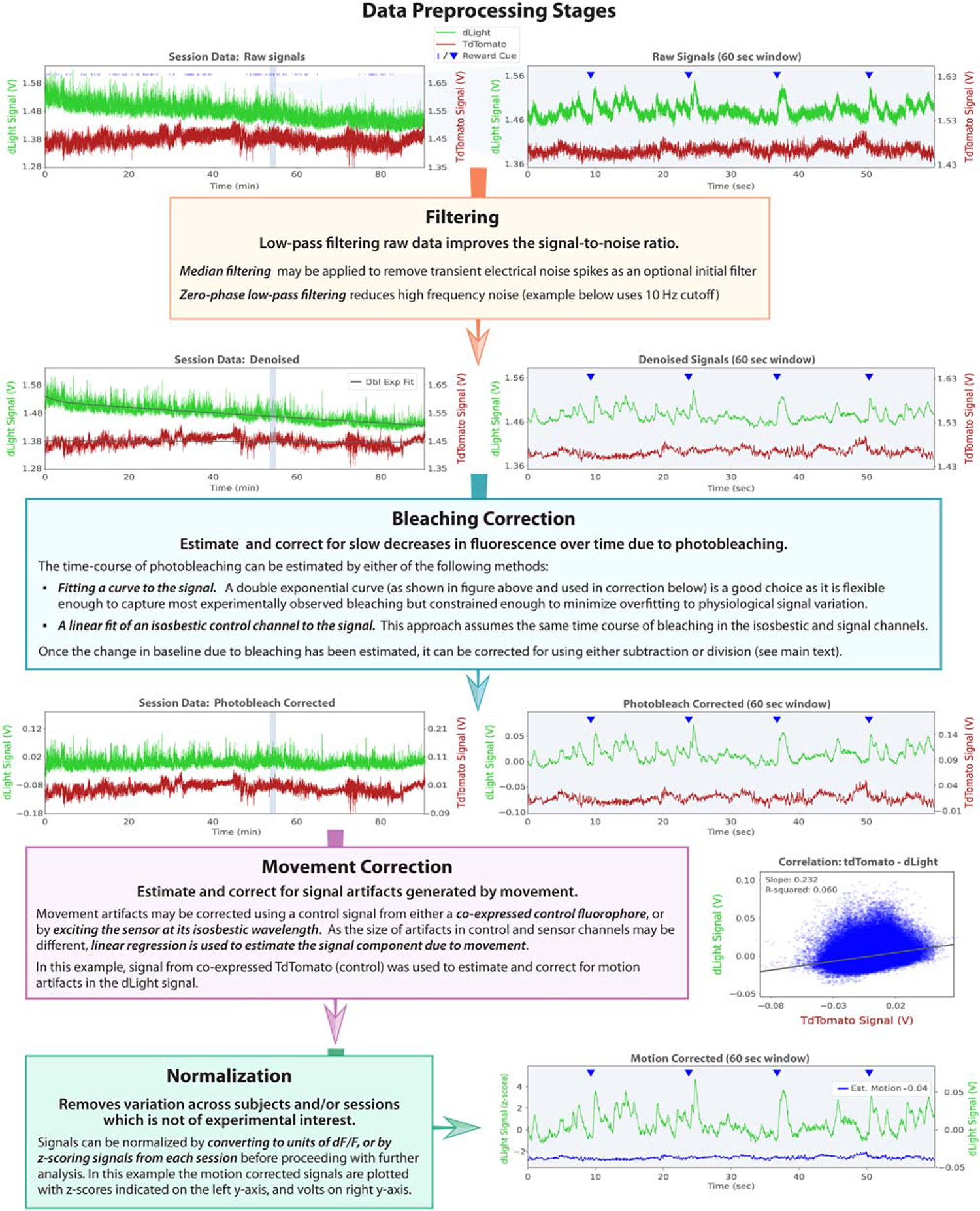
Data preprocessing stages Schematic diagram with example data describing the principal data preprocessing workflow used to remove noise and artifacts from raw photometry signals and convert them into appropriate units for comparison across sessions and subjects. Each box describes the function and methods for implementation of a discrete preprocessing stage. Example data are from photometry recordings targeting the nucleus accumbens core of wild-type C57BL/6 mice co-expressing dLight1.1 (pAAV5-CAG-dLight1.1) and a tdTomato control (pssAAV-2/5-hSyn1-chI-tdTomato-WPRE-SV40p(A)), during performance of a flexible, reward-guided decision-making task. Example input and output signals from each preprocessing stage (direction indicated by arrows) illustrate how the signals are modified at each step in the process. When multiple implementation options exist for a given stage, the method applied to the example data is indicated in the description. Where useful for illustrating the impact of a given preprocessing stage on both long and short timescales, both session data (approx. 90 min session, left plots) and a zoomed-in view of a 60-s window (right plots, with gray background) are plotted side-by-side. The onset of reward cues is indicated by blue ticks and triangles in session and 60-s window plots, respectively. dLight signals are plotted in green with units indicated on the left y axis, and tdTomato (control) signals plotted in red with units indicated on the right y axis. The double exponential fits of the denoised signals used to estimate photobleaching are overlaid in black. The correlation between tdTomato (control) and dLight signals at each time point in the session was used to estimate motion artifacts (right of movement correction box). The estimated signal due to motion artifacts (blue trace, offset by −0.04 for ease of viewing) is plotted along with the final motion-corrected signal. The normalized units (*Z* score) of the final, motion-corrected signals are indicated on the left y axis, and raw units (volts) on right y axis.

Preprocessing typically involves applying some or all of the following sequence of steps: filtering, bleaching correction, movement correction, and normalization. The effects of each step on sample data are presented in Figure 3. The code and example data for the pre-processing steps shown in Figure 3 are available as an IPython notebook; see the data and code availability statement.

#### Filtering

In most experiments, the kinetics of the indicator are slow relative to the sampling rate the recording system is capable of. Meaningful physiological signals are therefore only present in the low-frequency components of the recorded signal, whereas noise is present at all frequencies. The S/R ratio can then be improved by low-pass filtering. The optimum low-pass cutoff frequency will depend on the indicator; for example, 2–10 Hz is typically used for GCaMP6f and dLight1. Zero-phase filters, which change the amplitude but not the phase of frequency components, avoid distorting the signal. This can be implemented by functions like the Matlab/Scipy function *filtfilt*, which filters the signal first in the forward, then the reverse direction, canceling out phase shifts.

The high-gain amplifiers in photodetectors can pick up electrical noise, and some noise sources such as mobile phones can result in large amplitude, short-duration noise spikes. If present, these artifacts can often be largely removed by median filtering using a window ~5× longer than the duration of the noise spikes prior to any other filtering.

#### Bleaching correction

Photobleaching—a reduction in fluorescence over prolonged exposure to light—occurs both to the fluorescent indicator being measured and to autofluorescence from optical hardware and brain tissue. Bleaching of the indicator reduces the baseline level of the signal and the amplitude of physiological signal variation, whereas bleaching of the autofluorescence reduces only the baseline signal.

One approach to bleaching correction exploits the fact that bleaching occurs on a slow timescale relative to most physiological processes of interest. Therefore, the time course of bleaching can be estimated from the slowest components of the signal, either by filtering or curve fitting. The challenge is to provide enough flexibility in the estimate to capture the dynamics of the bleaching, but no more than necessary to avoid overfitting to physiological signal. Fitting a double exponential decay is a good compromise, as a single exponential can be too restrictive, given that different sources of fluorescence may bleach with different timescales, although filtering or fitting more complex curves could easily overfit.

Once the time course of bleaching has been estimated, it can be corrected for either by subtraction or division from the raw signal. These two options reflect different assumptions. If the bleaching is dominated by autofluorescence, then it will affect the baseline but not the amplitude of physiological variation, so should be corrected by subtraction. If bleaching is dominated by the indicator, then it will affect both baseline and signal amplitude and should be corrected by division. We are not aware of any systematic characterization of this and different studies use subtraction^102^ or division^50,103^ for photobleaching correction. Note that if division is used, this converts the signal into units of dF/F (see normalization below).

Another approach to estimating the time course of bleaching is to use an isosbestic control channel, which should not be affected by physiological signal variation (see section choice of sensor-specific controls). One caveat to this approach is that the relative contributions of autofluorescence and indicator fluorescence to the isosbestic and signal channels are likely to be different, as shorter wavelength isosbestic illumination typically excites more autofluorescence, and is less efficient at exciting the indicator than light at the peak of the excitation spectrum. This could potentially cause the time course of bleaching to differ between control and signal channels.

In behaviors that have a discrete trial structure, a third approach is to estimate the baseline separately for each trial (for example, from the signal in the preceding inter-trial interval) and subtract this from the signal on that trial. This approach should be used with caution during behavioral experiments because the level of physiological signal during the inter-trial interval may vary meaningfully across trials, meaning that trial-by-trial baselining may give a misleading picture.

#### Movement correction

Physical movement of the animal can generate signal variation through movements of the brain relative to the optic fiber or changes in light transmission due to the movement of connectors, rotary joints, or patch cords. Movement artifacts are minimal (although not necessarily eliminated) in head-fixed preparations, but in freely moving experiments, care must be taken to either verify movement artifacts are negligible or correct for their effect on photometry signals. As movement artifacts occur on a similar timescale to physiological signals, they cannot be separated using filtering; instead, they must be estimated using a separate control channel. The two options commonly used are as follows: (1) co-expression of a control fluorophore (e.g., red fluorescent protein) with different excitation and emission spectra from the indicator or (2) exciting the indicator at its isosbestic wavelength where fluorescence is independent of the physiological signal.

The size of movement artifacts relative to baseline fluorescence will in general be different between the signal and the control channel (see section choice of sensor-specific controls). It is therefore necessary to scale the movement signal from the control channel to match movement artifacts in the signal channel. This is typically achieved using linear regression to predict the signal channel from the control channel, with the rationale being that the component of the signal that can be predicted is movement artifact. This assumption will not be perfect as movement artifacts may be partially correlated with physiological signals but is a reasonable approximation. However, it will not hold if variation in the movement channel is dominated by signal bleed-through or if the common variation in both channels is dominated by photobleaching. For this reason, photobleaching correction should be done prior to, and independently from, movement correction, and it is important to verify that any signal bleed-through in the movement control channel is small relative to movement artifacts. This can be done by plotting the average movement channel response to behavioral events that generate large signal transients (see, e.g., Figure 2).

#### Normalization

In most experiments, it is necessary to combine data across multiple sessions and/or subjects. This is complicated by the fact that indicator expression may vary both between subjects and over the course of a multi-day experiment. Additionally, different intensities of excitation light may be used for different sessions, different hardware setups may vary in how efficiently they convert emitted light to signal and level of autofluorescence, and these hardware properties may change over time, particularly if components are replaced. Normalizing the data to remove variation across subjects and time points that are not of experimental interest is therefore desirable, and there are various methods available.

One approach is to convert signals into units of dF/F, i.e., the change in fluorescence signal divided by the baseline signal level. This is widely used in two-photon calcium imaging where the baseline fluorescence is closely related to the amount of indicator present^104^ but may be less effective in photometry due to autofluorescence. The relative contribution of indicator fluorescence and autofluorescence will be different between the signal (dF) and baseline (F), with the signal hopefully dominated by changes in indicator fluorescence, but the baseline potentially includes a large contribution from autofluorescence. Computing dF/F will therefore not necessarily correct accurately for different levels of indicator expression or differences in autofluorescence across setups, although it will correct for different levels of excitation light or efficiency in converting emitted light to signal.

Another approach is to *Z* score the signal for each session, i.e., subtract the mean and divide by the standard deviation. This will remove the influence of any factors that either scale the signal size (e.g., differences in indicator expression or excitation light intensity) or affect the baseline (e.g., the level of autofluorescence) between sessions. The limitation is that this may remove variation of experimental interest, such as changes in signal across learning over multiple sessions or fixed trait differences between subjects that might reflect the genetic manipulation of a mechanism under test or a transgenic model of disease versus a control group. Such differences may, however, be resolvable in *Z*-scored signals if they manifest in the response to specific behavioral events/temporal epochs of interest.

An alternate approach to account for differences in expression levels of sensors across time, brain regions, or animals could come from the development of new sensors that employ a cpGFP-based indicator directly fused with a spectrally orthogonal fluorescent protein (e.g., a red fluorescent protein) to generate a ratiometric sensor. This approach has been proven to work in the case of a voltage sensor, where the red and green FPs were located on opposite sides of the cell membrane. In the case of GPCR-based sensors, achieving this would require careful re-design of the probe to ensure that the two FPs do not give rise to FRET upon sensor activation and that the properties of the sensor (e.g., surface expression, affinity, and dynamic range) are unaffected. A detailed characterization of the effect of this fusion on the properties of the indicator would be required. Sensors created in this way would be excitable at two separate wavelengths leading to two distinguishable emissions, only one of which (the green one) would be ligand-modulated, allowing for a ratiometric readout that would in theory be independent of inter-subject and inter-session variation. This approach also comes with the following caveats: (1) the two FPs may exhibit different bleaching kinetics; (2) if the red channel had a substantial contribution from autofluorescence or other background sources, its efficacy in normalizing the signal from the green channel would be impaired; and (3) multiplexing with a second red-shifted indicator would not be possible. Currently, the existing sensors for obtaining absolute measures include FRET- and FRET-FLIM-based sensors (see section sensor selection for *in vivo* photometry), although they require specialized equipment (see section advanced hardware features).

#### Combining processing steps

A preprocessing approach widely used with isosbestic control channels (e.g., Mohebi et al.^50^ and Saunders et al.^103^) combines bleaching correction, movement correction, and normalization into a single operation. The control channel is first fit to the signal using a least-squares linear fit, and then, the processed signal is calculated by subtracting the fitted control from the signal, then dividing by the fitted control. The rationale is that the subtraction corrects for movement artifacts and changes in baseline due to photobleaching, and then the division corrects for changes in signal amplitude due to photobleaching and converts to dF/F. Combining movement and photobleaching correction in this way implicitly assumes that the relative size of movement artifacts between signal and control channels is the same as the relative size of photobleaching artifacts between the two channels. This assumption will in general not hold exactly, as the relative contributions of autofluorescence and sensor fluorescence will be different for isosbestic and sample channels, and these two sources of fluorescence will be differentially affected by movement and bleaching (see above). In practice, variation in isosbestic control channels is typically dominated by photobleaching; hence, this will determine the linear fit to the signal, prioritizing the accuracy of photobleaching correction above that of movement correction.

We are not aware of any systematic quantification (e.g., using a non-ligand-binding control sensor) of how effectively different control channels and preprocessing methods correct for artifacts in photometry signals, but this would be a valuable contribution to the literature.

### Analysis and statistical testing

There are many ways to analyze photometry signals. The most appropriate analytic approach will be determined by the structure of the experiment and the experimental questions being tested, which will determine the relevant signal comparisons to be made. Here, we describe approaches that have been used successfully and indicate the conditions under which they may, or may not, be suitable.

As with any experimental method, appropriate experimental design is necessary to ensure that the effect of different variables of interest can be differentiated from each other and possible confounds. A photometry-specific consideration is that changes in signal on a very slow timescale may be hard to conclusively differentiate from imperfect photobleaching correction. It is therefore good practice to ensure that different experimental conditions of interest are distributed evenly across the session.

#### Event-aligned analysis

Aligning photometry signals to an event of interest, such as reward presentation or omission, and averaging the data by trial type are widely used approaches to visualize how the signal is modulated around events. This can be followed by summary statistics, such as comparing peak signal amplitude or measuring the area under the curve (AUC) for a defined interval. It is important to note that the latter is sensitive to the selected time window, and therefore, this kind of analysis requires principled prior assumptions based on the nature of the photometry signals.

Many behavioral tasks include multiple events of interest. If events happen at fixed times (e.g., auditory cue followed by a fixed delay followed by a reward), time locking to one event will inherently time lock to the other events. However, this is not the case when trials are self-paced by the subject or variable intervals are imposed. In such cases, different approaches have been used as follows: (1) separately align the signal to each event of interest or (2) align to all the events in a trial by time-warping the signal between events. Time-warping is a useful method to visualize the response to all events across a trial (e.g., trial initiation, choice, and outcome) in decision tasks^102,105^ and can also be used to account for the variable duration of spontaneous behaviors.^106^ However, it is necessary to confirm that time-warping the signal does not introduce artifacts in the data, by separately aligning to the self-paced events.

#### Linear regression

**Figure 4.**
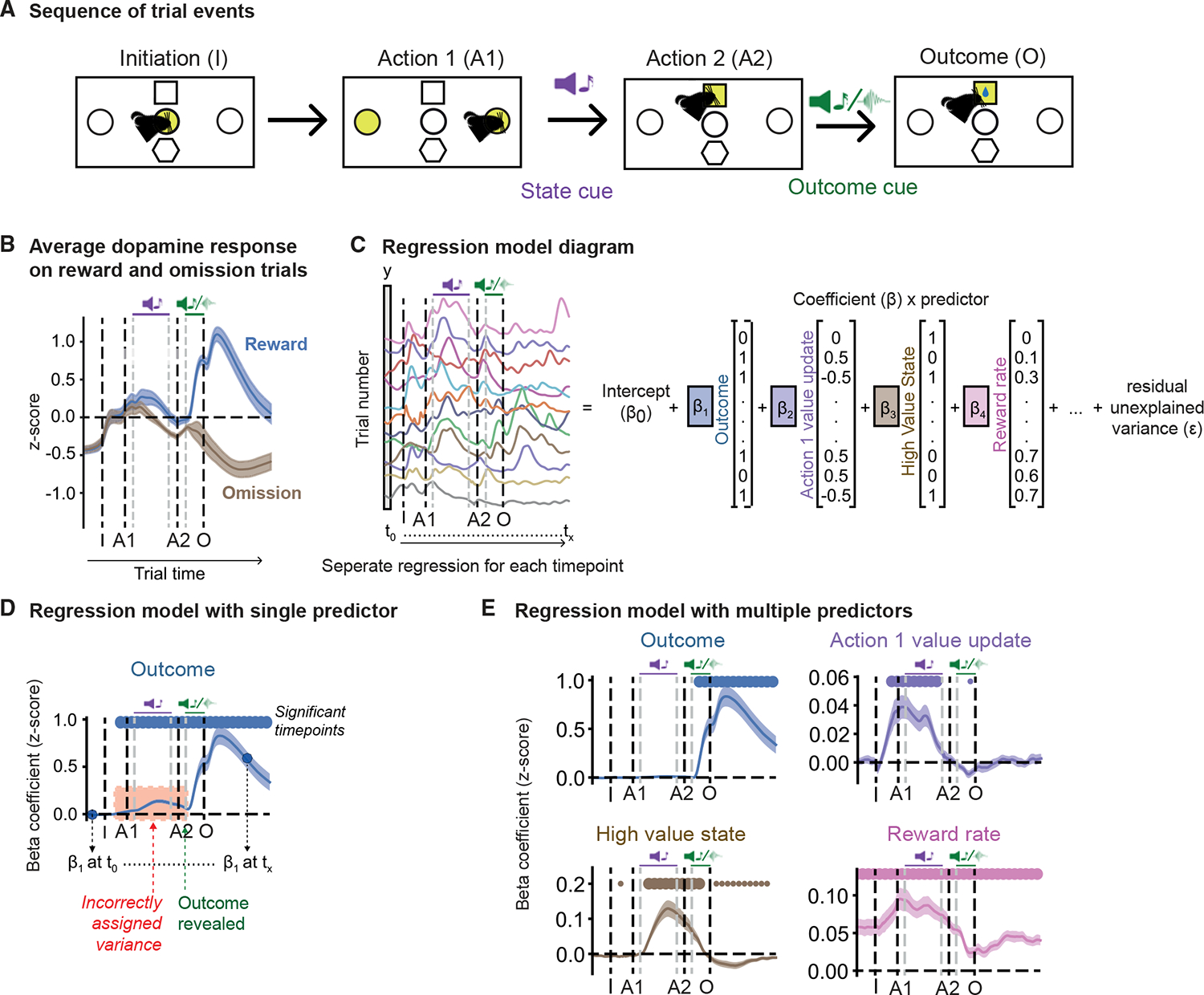
Analysis of photometry data using linear regression Analysis of photometry signals using linear regression, illustrated using calcium activity (GCaMP6f) in ventral tegmental area dopamine neurons during a multistep decision making task from Blanco-Pozo et al.^102^ (A) Diagram showing the sequence of events on each trial. Signals were aligned across trials by time-warping the activity to align the times of the trial events to match the median timing across trials. (B) Mean *Z* scored dopamine activity across the trial, split by outcome (reward or omission); shaded area indicates cross-subject standard error of the mean. Note that the average dopamine signal on reward and omission trials separates before the outcome cue (green bar), i.e. before information about the outcome was available, due to the influence of subject’s reward expectation. (C) Schematic of the regression model. A separate linear regression was run for each timepoint in the trial aligned activity. Each regression models the activity at that timepoint as weighed sum of predictors, where each predictor has a β coefficient that indicates how strongly and with what sign the predictor explains variance in the activity. The predictors take different values from trial-to-trial but the same value for all timepoints in a given trial. (D and E) Plotting the time-course of β coefficients for a given predictor indicates when in the trial the predictor explains variance. Separate regression analyses were run for each subject; traces show cross-subject mean, shaded areas cross-subject standard error. Dots above the traces show time-points where the β coefficient was significantly different from 0, assessed using a t test on the cross-subject distribution with Benjamini-Hochberg correction for comparison of multiple timepoints. In (D), the regression analysis included only a single predictor coding for the trial outcome. This regression has significant positive coefficients for the outcome predictor before information about the outcome is available (red shaded area). This is because the subjects’ expectation of reward drives variation in the signal which is correlated with the trial outcome (as seen in B). (E) By including additional predictors in the regression, which in this example relate to the value of the actions taken, state reached on the trial, and the recent rate of rewards over the past 8 trials (see Blanco-Pozo et al.^102^ for details), we can both resolve the influence of these different behavioural variables at different timepoints and remove the spurious loading on the outcome predictor before the outcome cue, as variance in the signal due to reward expectation is now captured by the other predictors.

In situations where multiple behavioral variables are expected to influence the signal, particularly where these may be correlated (e.g., reward prediction and trial outcome, see Figure 4), a multiple linear regression provides a simple but powerful approach to quantifying which behavioral variables account for signal variation at different time points. This method has been extensively used to analyze neural data, including fMRI,^107,108^ electrophysiology,^109–112^ one- and two-photon^113–117^ excitation microscopy, and, more recently, photometry data.^14,25,102,118^ In Python, for example, linear regression can be implemented using the sklearn.linear_model module from the scikit-learn library.^119^

The general approach is to model variation in the signal as a linear combination of a set of predictors generated from behavioral variables of interest. An important choice in implementing a regression analysis of photometry data is how the varying influence of variables over time is modeled. For data with a discrete trial structure, one approach is to run a separate regression analysis at each time point across the trial (Figure 4B), using predictors that take the same value for all time points on a given trial but vary from trial to trial. Such predictors are usually categorical or binary and indicate what happened on each trial. For example, the trial outcome could be coded as a binary *reward* predictor, set to “1” if a reward was obtained or “0” if no reward was obtained (Figure 4B). This approach yields a β coefficient for each predictor at each time point across the trial, such that plotting the βs for a given predictor gives a time series showing when, in which direction, and how strongly that predictor explains variance in the signal.

A second approach is to model all time points in a single regression analysis. As discrete behavioral events typically produce a temporally extended and delayed response in the photometry signal, the time course of the predictors associated with these events needs to capture this. This can be achieved by convolving the sequence of event times with one or more temporal basis functions designed to capture the expected time course of signal variation due to a single event, for example, a B-spline basis, as implemented by the *bs* and *patsy* packages in R and Python, respectively (see, e.g., Engelhard et al.^114^). Using a single basis function represents a fixed assumption about the time course of signal variation associated with each event, whereas using a set of basis functions (each with its own β) can model any time course that is a linear combination of those bases. Sometimes, the influence of a continuously changing variable, such as the subject’s speed of movement, may be of interest. If a linear relationship is expected between the variable value and the photometry signal, the variable may be used directly as a predictor, potentially with a lag to capture the delay between changes in the variable and the signal.

A potential caveat to be aware of in any regression analysis is that variance can be incorrectly modeled in situations where there are correlations between variables and not all of the variables are included in the model. For example, Figure 4C shows an analysis of dopamine activity in a probabilistic reward-guided decision-making task, using the approach of running separate regressions at each time point to obtain a time -series of β coefficients, in which the only predictor included was the trial outcome (rewarded or not). This appears to show highly significant positive βs at time points *before* the outcome was revealed, when the outcome of that trial was not known and so should not be able to influence the recorded signal. This occurs because dopamine activity at these time points is influenced by the subject’s prediction of the outcome, which is correlated with the actual outcome; the spurious loading disappears when additional predictors capturing the subject’s outcome prediction is included (Figure 4D).

Another potential issue in regression analyses, termed Collin-earity, occurs where one predictor is highly correlated with another predictor or a linear combination of other predictors. This makes the fit very sensitive to small changes in the data, as different βs yield very similar predictions for the signal. Regularization—i.e., adding an additional term to the cost function that penalizes large βs—can break this degeneracy but should be used with caution as the fitted βs can then reflect the effect of regularization rather than structure in the data itself. L1 regularization (also called Lasso) promotes sparseness in the coefficients, whereas L2 regularization spreads the influence of each coefficient more evenly. If regularization is used, it is important that regressors are standardized or centered at 0; hence, the penalty used is the same across all the regressors. This also allows comparison of the magnitude of the influence on the signal across regressors.

#### Statistical testing

Different methods can be used for statistical testing of any photometric measures. Typically, ANOVA or t tests with correction for multiple comparisons to control for false discovery rates are used. Alternatively, confidence intervals and bootstrapping may be used (see Jean-Richard-dit-Bressel et al.^120^).

For between-subject comparisons, it should not be assumed that data points are independent and identically distributed; incorrectly assuming so can result in inflated false positives. Mixed-effect models overcome these limitations by capturing dependencies in the data through their random effect structure.^121^ In R, mixed-effect models can be implemented using *lmer* or *afex* packages. Some drawbacks of mixed-effect models are that they are computationally costly and can have problems with convergence. Moreover, it has not yet been resolved what the best approach to define the random effects structure should be.^122–124^ Although future research should help clarify this, a simple workaround that has been shown to produce comparable results is to perform two-stage summary statistics^125,126^: first, a regression model is run for each subject separately, followed by a second stage in which all individual means (or the individual means combined with within-subject variances) are used to produce estimates of between-subject variance and enable group inferences. This approach is widely used with fMRI data (see Mumford and Poldrack^127^).

## LOOKING FORWARD

Over the past decade, fiber photometry has become an established, core technique that, coupled with developments in behavioral profiling, has the potential to revolutionize our understanding of how dynamic changes in neurotransmission relate to rich repertoires of animal behavior. Here, we summarize the opportunities that fiber photometry currently offers, describe the new tools for fiber photometry that are in development, and consider what may be achieved in the future.

### Current opportunities

A truly groundbreaking opportunity to understand the neurobiological mechanisms of neural communication and causal mechanisms of behavior comes from combining photometry with other techniques. Manipulating neural activity with opto-genetics while simultaneously recording the consequences via photometry provides information that was completely unattainable with previous methods.^32–34,38,39,52,128^ Successful examples include activation or inhibition (at the level of soma or terminals) of one cell type (e.g., dopamine, orexin, or oxytocin neurons), and simultaneous measurement of modulator release from those same neurons.^129^ Alternatively, the influence of activity in one type of neuron on the release of a different neuromodulator can be determined, e.g., the effect of GABAergic activity on serotonin release^117^ or the effect of cholinergic activity on dopamine release.^130^ In addition, by combining two spectrally resolvable sensors researchers can determine temporal correlations in the dynamic activity of up to two distinct aspects of neural activity, for example, dopamine release in the nucleus accumbens and the activity of accumbens D1-expressing spiny projection neurons or intracellular PKA activity in D1 or D2-expressing spiny projection neurons.^25^

Combining photometry with other methods can also be used to improve experimental approaches. Photometric readouts of manipulations like optogenetics or focused ultrasound can facilitate calibration of stimulation parameters to understand the effects on different cell types^131^ or more accurately match endogenous release patterns.^132^ The recapitulation of behaviors using neuromodulation that is optimized using photometry provides validation that photometrically recorded biosensor signals are physiologically relevant. For example, optogenetic stimulation calibrated to photometrically reward responses is sufficient to produce conditioned place preference.^133^

The relative simplicity of photometry and the small size of optic fibers make it ideally suited to use in combination with other (non-fluorescence-based) recording techniques. This already has been used to better understand the effect of signaling in defined cell types and neurotransmitter release on blood-oxygen-level-dependent (BOLD) fMRI signals^134–136^ and, conversely, in combination with electrophysiology, to identify the potential source of calcium fluctuations recorded in striatal neurons using photometry.^137^

### Emerging directions

A newly emerging topic of research dependent on fiber photometry is the identification of the different timescales over which neurotransmitters and neuromodulators act and how these fluctuations on different timescales relate to the temporal dynamics of different behavioral actions or behavioral states. In contrast to microdialysis, which lacks sufficient temporal resolution to pick up sub-minute fluctuations, or cyclic voltammetry, which typically is better suited to detecting rapid but not sustained changes in neurotransmitter levels, fiber photometry can quantify events occurring both on sub-second timescales tied to specific task events and sustained changes that are modulated over many minutes.^10,102,138,139^

While opening up this fascinating new dimension, it is imperative to interrogate slower changes carefully, given the potential for these to be influenced by artifacts such as photobleaching or, in reward-guided tasks, confounded by endogenous changes such as satiety. Moreover, signals of interest may be contaminated by changes in pH^140–143^ and hemodynamics,^15–17^ which will usually fluctuate on slower timescales. It will therefore be important to implement appropriate experimental designs to obviate some confounds (e.g., by decorrelating anticipated slower changes from drift over the session), and where necessary, test sensors with ligand-binding site mutations. Further work characterizing the relationship between slower fluctuations resolved with fiber photometry and gold-standard measurements such as microdialysis will also be informative.^10^

On even longer timescales, fiber photometry provides an unprecedented opportunity to conduct longitudinal studies. Both the expression of biosensors and the functioning of implanted optical fibers can be relatively stable across months. Therefore, changes in signals can be tracked over extended time periods as animals learn complex tasks and adapt to different environmental contingencies, and as the brain circuits mature, cellular aging occurs, and disease models develop. Again, analytic methods must prevent the contamination of long-term changes by artifactual drifts. Developments of fluorescence lifetime-based measurements and sensors hold promise for improving longitudinal studies as their readouts are independent of sensor expression levels,^4,64,144^ although currently setups are often bespoke and therefore come at a cost of increased technical complexity.

Another potential avenue for development is to harness molecular specificity to restrict the location or functional activity of biosensors to select subcellular compartments. Currently, commonly used membrane-bound biosensors are not spatially confined to the synapse; hence, differentiating between synaptic and extrasynaptic signals requires high-resolution imaging in combination with synaptic markers. However, if the location or function of membrane-bound biosensors could be restricted or excluded from synapses generally or synapses of certain cell types, then fiber photometry could be used to gather more detailed information about neurotransmitter signaling.

Across different scales of time and space, the versatility of photometry offers unprecedented opportunities to broaden research into the link between neural dynamics, interacting neurotransmitters, and complex, naturalistic behavior. Photometry has already successfully been used to track cell-type-specific effects of ethologically relevant behaviors such as social interaction, maternal behavior, mating, and feeding^3,62,145–151^ and help dissect transitions between different action motifs during behavioral sequences.^6,106^ As technology advances to allow wireless^60^ and even implantable photometry systems,^152^ alongside innovations in behavioral monitoring of groups of animals in semi-natural contexts,^153^ the capability to tackle deep questions about the relationships between the environment, neural activity, and complex behavior in health and disease will become ever more tractable.

Progress in understanding universal brain-behavior relationships will be accelerated by the ability to use photometry across a range of model species, including birds^154^ and non-human primates,^155^ and rodents. However, although photometry should be equally suited to any mammalian or avian species when selecting sensors and promoters, testing must be performed to ensure that expression is robust and stable. It cannot be *a priori* assumed that what works in a mouse will necessarily translate directly even to other rodent species.

### Conclusions

The ability to run photometry experiments at scale and increasingly low cost as more open-source hardware becomes available has the potential to be transformative for neuroscience. First, it can help democratize research into brain-behavior relationships, facilitating a wide range of groups to ask creative questions, and not just those in the most highly resourced institutions.^156^ Second, it will support increased reproducibility, which in turn will facilitate further technical and theoretical innovations. However, continued progress depends crucially on the community being cognizant of the potential limitations of the method, understanding how best to design experiments within these constraints, and collect and interpret the data appropriately. We hope this guide will provide the signposts to facilitate this.

Fiber photometry continues to evolve rapidly. The catalog of sensor domains is expanding, including hard-to-study molecules such as neuropeptides. New fluorescent reporter domains and detection modalities are also being developed to provide better quantification of ligand concentration or with longer wavelengths for better spectral resolution in multiplex experiments. Also, at longer wavelengths, excitation light is less scattered and absorbed in tissue, potentially increasing the depth of signal collection. Recent innovations in hardware will make capabilities like multi-site and multi-sensor recordings ever more accessible. Combined, these developments will allow investigators to ask new questions, such as how does the release of one transmitter alter the activity or the release of another molecule locally, or simultaneously across a whole brain structure, or at multiple nodes within a circuit? What roles do molecules play in coordinating activity within and between brain regions, and how does that coordination relate to behavior? Because of all the described features, fiber photometry will continue to grow in popularity as a go-to tool to monitor the rich dynamics of biomolecules and physiological events over different temporal scales during complex behavior.

## Figures and Tables

**Table 1.** Comparison of typical features of *in vivo* detection methods

	Fiber photometry	E-Phys	FSCV	Microdialysis	Mini-scope	Two-photon scope

Cell specificity	yes	limited^a^	no	no	yes	yes
Molecular specificity	yes	no	yes^b^	yes	yes	yes
Spatial resolution (cell or subcellular)	multiple cells/processes	cellular	regional (~100 mm)	regional (~1 mm)	cellular	subcellular
Temporal resolution	10–100 ms^c^	<1 ms	100 ms	10 min	10 ms	10 ms
Setup cost (thousands of US$)^d^	$5-$25	$5-$100	$10-$30	$8-$65^e^	$5-$250	$125-$300

The values provided are in the typical practical range used and do not represent theoretical limits of the techniques.

a Cell specificity is limited by the efficacy of cell-sorting based either on waveform, which is more reliable for some cell types than others, or by targeting a light-sensitive opsin to a particular cell type. The latter requires a more complex surgery, an integrated optical and electrophysiology system and is prone to variability in yields across animals and cell types.

b Molecular specificity depends on the uniqueness of the electrochemical profile of the molecule of interest within the sampling environment. Relatively few molecules have been validated.

c Temporal resolution is limited by the temporal dynamic performance of the sensor, which varies.

d Approximate costs are based on a single setup and range from custom built equipment using open-source designs, materials and software to preassembled commercially available complete packages. Costs included are specifically for the technique listed, they do not include common core equipment such as a stereotaxic device for surgery etc.

e The low end is for sample collection and preprocessing only, assuming outsourcing sample analysis. The upper end includes equipment for analytical chemistry which can range depending on the number of different analytes and the sensitivity required.

**Table 2.** List of genetically encoded sensors used for *in vivo* fiber photometry recordings of neural activity

Sensor name	Ligand	Sensing domain	Reporter domain	Isosbestic wavelength	Control sensor	*In vivo* pharmacology	Photometry validation	Photometry application

GCaMP6s,m,f JGCaMP7s,f,b JGCaMP8	Ca^2+^	CaM/M13	cpGFP	420–430 nm	N/A	N/A	Chen et al.,^2^ Dana et al.,^20^ Zhang et al.^42^ and Meng et al.^43^	Patriarchi et al.,^33^ Meng et al.,^43^ Pisansky et al.^44^, and Formozov et al.^45^
JGCaMP7c	Ca^2+^	CaM/M13	cpGFP	350 nm	N/A	N/A	Dana et al.^20^	-
JRGECO1a, 1b	Ca^2+^	CaM/M13	cpmApple	400–420 nm	N/A	N/A	Dana et al.^21^	Patriarchi et al.^32^ and Meng et al.^43^
R-CaMP2	Ca^2+^	CaM/cckp	cpmApple	420–440 nm	N/A	N/A	Inoue et al.^46^	Kim et al.^47^
XCaMP-B	Ca^2+^	CaM/cckp	cpBFP	N/A	N/A	N/A	Inoue et al.^48^	-
XCaMP-G	Ca^2+^	CaM/cckp	cpEGFP	350–380 nm	N/A	N/A	Inoue et al.^48^	-
XCaMP-Y	Ca^2+^	CaM/cckp	cpVenus	400 nm	N/A	N/A	Inoue et al.^48^	-
XCaMP-R	Ca^2+^	CaM/cckp	cpmApple	440 nm	N/A	N/A	Inoue et al.^48^	-
NIR-GECO2	Ca^2+^	CaM-RS20	mIFP	400–440 nm^a^	N/A	N/A	-	Formozov et al.^45^ and Qian et al.^49^
dLight1.1 dLight1.2 dLight1.3b	DA	human DRD1	cpGFP	N/A	dLight-ctr	SCH-23390 (antagonist)	Patriarchi et al.^32^	Ejdrup et al.,^10^ Ma et al.,^17^ Lee et al.,^25^ Mohebi et al.,^50^ and Jong et al.^51^
GRAB_DA2h,2m,_ GRAB_DA1h,1 m_	DA	human DRD2	cpGFP	440 nm	GRAB_DA-mut_	eticlopride (antagonist)	Sun et al.^34,52^	-
RdLight1	DA	human DRD1	cpmApple	N/A	N/A^b^	SCH-23390 (antagonist)	Patriarchi et al.^33^	-
rGRAB_DA1h,1m_	DA	human DRD2	cpmApple	400–420 nm	rGRAB_DA-mut_	eticlopride (antagonist)	Sun et al.^34^	-
GRAB_NE1h,1m_	NE	human Alpha2AR	cpGFP	N/A	GRAB_NE-mut_	yohimbine (antagonist)	Feng et al.^35^	-
nLightG	NE	sperm whale Alpha1AR	cpGFP	428 nm	N/A^b^	trazodone (antagonist)	Kagiampaki et al.^40^	-
nLightR	NE	sperm whale Alpha1AR	cpmApple	450 nm	N/A^b^	trazodone (antagonist)	Kagiampaki et al.^40^	-
GRAB_eCB2_	eCBs	human CB1R	cpGFP	415 nm	GRAB_eCB-mut_	N/A	Dong et al.^53^	-
GRAB_ATP1.0_	ATP	human P2Y1R	cpGFP	435 nm	GRAB_ATP-mut_	N/A	Wu et al.^54^	-
GRAB_HA1h,1m_	HA	human H4R; waterbear H1R	cpGFP	420 nm	GRAB_HA-mut_	JNJ-7777120 (GRAB_HA1h_ antagonist)	Dong et al.^55^	
GACh_1.0_ GACh_2.0_ GRAB_Ach3.0_	Ach	human M3R	cpGFP	384 nm + 434 nm	GRAB_Ach3.0-mut_	scopolamine (antagonist)	Jing et al.^36,56^	
iAchSnFR	Ach	PBP	cpGFP	400–425 nm	iAchSnFR_NULL_	N/A	Borden et al.^31^	-
GRAB_5HTI.0_	5HT	human 5HT2CR	cpGFP	N/A	GRAB_5HT1.0-mut_	metergoline (antagonist)	Wan et al.^57^	-
PyschLight2	5HT	human 5HT2AR	cpGFP	N/A	PsychLightO	ketanserin (antagonist)	Dong et al.^41^	-
iSeroSnFR	5HT	PBP	cpGFP	N/A	N/A	N/A	Unger et al.^58^	-
GRAB_Adoi.o_	Ado	human A2AR	cpGFP	N/A	GRAB_Ado-mut_	N/A	Peng et al.^59^ and Wu et al.^60^	Peng et al.^59^
OxLight1	OXA/OXB	human OX2R	cpGFP	432 nm	OxLight-ctr	suvorexant	Duffet et al.^38^	-
NOPLight1	N/OFQ	human NOPR	cpGFP	435 nm	NOPLight-ctr	J-113397	Zhou et al.^61^	-
Mtria_ot_	oxytocin	medaka OTR	cpGFP	N/A	MTRIA_OT-mut_	N/A	Ino et al.^39^	-
Grab_ot_	oxytocin	bovine OTR	cpGFP	425 nm	GRAB_OT_-mut	atosiban	Qian et al.^62^	-
iGluSnFR	Glu	PBP	cpGFP	425 nm	iGluSNFR(mut)	N/A	Marvin et al.^28,29^	Patriarchi et al.^33^
iGABASnFR	GABA	PBP	cpGFP	425 nm	N/A	N/A	Marvin et al.^30^	-
cADDis	cAMP	EPAC cAMP binding domain	cpGFP	N/A	N/A	N/A	Tewson et al.^63^	Lutas et al.^22^
G-Flampi	cAMP	CNBD (bacterial *MlotiK1* channel)	cpGFP	350 nm	G-Flamp1-mut^c^	N/A	Wang et al.^23^	
FLIM-AKAR	PKA activity	PKA substrate peptide + FHA domain	FRET-FLIM between mEGFPΔ and cpsREACH	N/A	AKAR-T391A: (phospho-dead mutant)	N/A	Lodder et al.^24^	Lee et al.^25,64^
MacQ-mCitrine	voltage	*Ace* rhodopsin VSD	electrochromic FRET (mCitrine-opsin)	N/A	N/A	N/A	Gong et al.^65^	Marshall et al.^26^
Ace2N-4AA-mNeon	voltage	*Ace* rhodopsin VSD	electrochromic FRET (mNeonGreen-opsin)	N/A	N/A	N/A	Gong et al.^65^	Marshall et al.^26^
VARNAM	voltage	*Ace* rhodopsin VSD	electrochromic FRET (mRuby3-opsin)	N/A	N/A	N/A	Kannan et al.^27^	

CaM, calmodulin; cpGFP, circularly-permuted green fluorescent protein, DA, dopamine; DRD1, dopamine D1 receptor; DRD2, dopamine D2 receptor; GRAB, G-protein-coupled receptor activation-based; NE, norepinephrine; Alpha2AR, Alpha-2 adrenergic receptor; CB1R, cannabinoid receptor type-1; eCBs, endocannabinoids; P2Y1R, purinergic P2Y1 receptor; Ado, adenosine; OXA/OXB, orexin-A/orexin-b; N/OFQ, nociceptin/orphanin-FQ peptide; H1R, human histamine type-1 receptor; H4R, waterbear histamine type-4 receptor; HA, histamine; M3R, muscarinic M3 receptor; Ach, acetylcholine; 5HT2CR, 2C-type serotonin receptor; A2AR, adenosine 2A receptor; OX2R, orexin type-2 receptor; NOPR, nociceptin/orphanin-FQ receptor; OTR, oxytocin receptor; Glu, glutamate; EPAC, exchange protein activated by cAMP; CNDB, cyclic nucleotide-binding domain; FHA, Forkhead-associated phosphopeptide-binding domain.

a Based on spectral characterization of NIR-GECO1.

b Although not described in the respective original publications, control sensors for RdLight1, nLightG, and nLightR are available upon request from the Patriarchi laboratory.

c This control sensor has been described, but not validated using *in vivo* fiber photometry.

## Data Availability

Sample data, code, and additional information for Figure 3 are openly available in the GitHub repository: https://github.com/ThomasAkam/photometry_preprocessing. https://doi.org/10.5281/zenodo.10103973.
